# Analysis of Reproductive Strategies and Immunological Interactions in *Batrachochytrium dendrobatidis*-Resistant Japanese Tree Frogs

**DOI:** 10.3390/ani15020154

**Published:** 2025-01-09

**Authors:** Ji-Eun Lee, Jun-Kyu Park, Yuno Do

**Affiliations:** Department of Biological Sciences, Kongju National University, Gongju 32588, Republic of Korea; jelee00@smail.kongju.ac.kr (J.-E.L.); pjk8578@kongju.ac.kr (J.-K.P.)

**Keywords:** amphibian, disease sensitivity, host-pathogen interaction, population sustainability, terminal investment

## Abstract

The amphibian chytrid fungus, *Batrachochytrium dendrobatidis* (*Bd*), has been implicated as an agent of acute declines in amphibian populations worldwide. East Asian amphibians have been coexisting with *Bd* for long periods and thus are considered resistant; among the many is the Japanese tree frog, *Dryophytes japonicus*. Our study focused *Bd* infection effects on reproductive behaviors and physiological parameters in *D. japonicus* as a function of better understanding the chronic effect of the disease on long-term population viability. During the peak breeding season, we captured 70 males and quantified the chorus size, calling behaviors, physiological states, innate immunity, and sperm quality of individuals. In a simple comparison, all parameters were not significantly different. However, in the NMDS analysis, we were able to confirm subtle trends in some items according to infection and correlations between several items. Importantly, sperm density and sperm vitality tend to increase with *Bd* infection load, suggesting increased reproductive effort following infection. Additionally, this analysis indicated that innate immunity was positively related to *Bd* infection intensities, indicating the activation of immunity upon infection. These findings indicate that *Bd*-resistant *D. japonicus* maintains reproductive capabilities and physio-logical stability despite *Bd* infection, likely due to a co-evolved immune system. The present work offers insight into how amphibian populations may have some endurance in the presence of *Bd* and points out the importance of studying resistant species as a means to understand long-term ecological effects. Our results suggest that resistance to *Bd* may not simply prevent cata-strophic declines but actively contribute to the dynamics of *Bd* prevalence in amphibian communities, and confer implications for conservation strategies.

## Simple Summary

We studied Japanese tree frogs (*Dryophytes japonicus*) resistant to *Batrachochytrium dendrobatidis* (*Bd*), a serious disease of worldwide amphibians. We compared calling parameters and physiological status related to reproduction with and without *Bd* infection in the field investigation. Although no significant differences were detected in all variables in the comparison results, some trends were found. As the *Bd* infection load increased, sperm quality tended to increase together, and innate immunity was also enhanced. As a result, species with *Bd* resistance can maintain or even increase their population size well under *Bd* infection. This could also serve as a mechanism by which species that have experienced population declines due to *Bd* can recover from declines when they later acquire *Bd* resistance.

## 1. Introduction

The amphibian chytrid fungus, *Batrachochytrium dendrobatidis* (*Bd*), is the causative agent of a rampant pandemic that has been implicated in the decline of amphibians across a wide range of habitats [1]. Severe declines due to *Bd* infection have been recorded mostly in Europe, Australia, and the Americas [2,3]. *Bd* causes hyperkeratosis of amphibian skin, ultimately reducing skin permeability, which leads to electrolyte imbalances [4]. This chytridiomycosis eventually causes heart failure and the death of the individual [5]. However, *Bd* infection is not considered fatal to amphibians in East Asia, since *Bd* strains and amphibians have historically coexisted for a long time in Asian regions such as the Korean Peninsula [6]. As evidence, many Asian amphibians have lower infection loads of *Bd* than amphibians from other continents [7,8,9]. For these reasons, Asian amphibian species are not considered appropriate model species for studying *Bd* susceptibility or are often ignored in studies assessing population status with *Bd* infection.

Although *Bd* virulence to amphibians remains high [9], some amphibian populations have been able to survive this serious disease, recover, develop immune defenses, and coexist with *Bd* [10,11]. Adaptation and co-existence with *Bd*, however, does not mean the end of research on this pathogen. Populations in countries that have recently experienced *Bd* infection and are currently facing severe population declines will gradually recover successfully and become more immune to *Bd*, as East Asian species have done through coevolution with *Bd* [7]. In simple terms, populations that have adapted to *Bd* represent the future of populations that have recently suffered severely from *Bd*. Therefore, studying species that have adapted to coexist with *Bd* can provide insights into the long-term impacts of *Bd* on populations. Amphibians in Korea are especially suitable for the study of population sustainability with continuously coexisting *Bd*, as *Bd* infection is common even among species with developed immune systems that can resist the disease [7]. When amphibians experience pathogen exposure, they may initiate resource shifts away from growth and reproduction either to clear or tolerate the infection [12,13]. This reallocation is critical because even if *Bd* infection is not lethal, it can impact population sustainability in various ways, from altering reproductive or survival behaviors to directly affecting reproductive capacity.

Previous studies showed that Japanese tree frogs (*Dryophytes japonicus*) that are resistant to *Bd* infection increase calling rate and duration following *Bd* infection, hereby increasing calling effort [14]. Increased calling efforts might attract females, hence facilitating the spread of *Bd*. This may be a strategy that favors reproduction over other life-history traits. Indeed, increased calling effort is energetically costly and may divert resources away from other physiological processes such as immunity [15]. A study on the Northern leopard frog (*Lithobates pipiens*), another resistant species, found that frogs infected with *Bd* had larger testes compared to uninfected frogs after eight weeks [16]. One possible explanation for this is that *Bd* infection truncates the reproductive lifespan of these frogs and is thus associated with “terminal investment”, where energy is directed toward reproduction at the expense of other life-history traits [17]. Accordingly, pathogens may reduce overt signs of disease but can lead to increased reproductive effort, which, in turn, influences life-history processes such as lifespan and reproductive capability. This may reduce the long-term survival of individual frogs and could affect population sustainability.

In contrast to predictions, several studies have found that the coexistence of *Bd* with highly susceptible species can maintain or even increase population sizes. One spatial modeling study suggested that populations of the highly *Bd*-susceptible Midwife toad (*Alytes obstetricans*) can grow despite high pathogen prevalence [18]. Similarly, seven years of population monitoring of southern Darwin’s frogs (*Rhinoderma darwinii*), also highly susceptible to *Bd* infection, showed that populations with high *Bd* prevalence had increased male reproductive effort, leading to higher recruitment and population growth despite high host mortality [19]. These findings also suggest that in *Bd*-susceptible species, compensatory mechanisms at the population level could offset the negative impacts of this pathogen. However, the detailed mechanisms of these responses, especially after adaptation to the *Bd* pathogen, remain very poorly understood. Further research will be required to identify whether physiological reproductive investment increases with the observed rise in reproductive effort. Understanding such a process might provide the key insights into individual and population-level responses to *Bd*, placing such knowledge in a critical position in framing conservation strategies.

With this study, we attempted to address how *Bd* infection impacts reproduction-related behaviors and physiology in the Japanese tree frog, *Dryophytes japonicus*, known to be resistant to *Bd* [14]. The focus was on male densities and calling behavior related to reproduction, steroid hormones, innate immunity, and sperm quality. These traits represent single units of response to disease, but variation in these traits could be correlated and thus interrelated. Therefore, we tried to identify associations among these components, not solely single responses, but ultimately in relation to infection load. This study will provide insights into the long-term sustainability of both the population and individual-level responses to *Bd* infection.

## 2. Materials and Methods

### 2.1. Field Investigation

**Figure 1:**
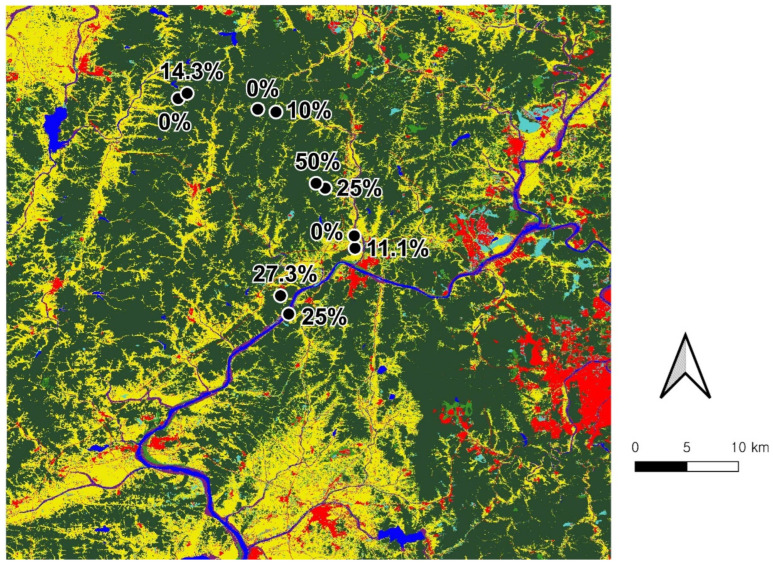
Sampling sites for Japanese tree frogs (*D. japonicus*) in paddy fields at 10 sites of Gongju-si. The *Bd* infection rate within each site is shown above each site.

We collected frogs from Gongju-si in mid to late May 2023, the peak breeding season of Japanese tree frogs. Japanese tree frogs are prolonged breeders, and their call components and physiological indices may change during the breeding season [20]. These factors were likely to vary within two weeks according to a previous study; thus, all experiments were completed within two weeks to exclude the breeding season effect. The survey was conducted simultaneously by two teams at one site, each consisting of two experts. The two teams worked together from 9:00 p.m. to 10:00 p.m., allowing them to collect nine frogs per site. We had found 10 sites where Japanese tree frogs were actively breeding through preliminary investigation, and conducted sampling at those sites. A total of 90 male frogs were collected across 10 sites, with similar characteristics among sites, including terraced paddy fields (Figure 1).

We searched for frogs in the field, recorded their calls, collected the recorded frogs, extracted saliva, and swabbed their skin. Afterwards, we transported the frogs to the laboratory, extracted blood. Blood-extracted individuals were euthanized in 5 g/L Tricaine methanesulfonate (MS-222, Sigma A5040) for dissection. MS-222 was adjusted to pH 7.0 with the addition of sodium bicarbonate. The frogs were euthanized by submerging their abdomens in the solution. Since 0.2 g/L to 0.5 g/L is the optimal concentration for amphibian anesthesia, we used 5 g/L MS-222 for about 5 min to humanely euthanize the adult frogs without stress [21,22,23]. We ensured that they had stopped breathing and that their hearts had stopped before collecting their testes by dissection. We selected a total of 70 frogs (6–9 per site) and used them for analysis. The animal experiment procedures were performed in accordance with the regulations and approval of the Experimental Animal Ethics Committee of Kongju National University (KNU_2023-07).

### 2.2. Recording and Analyzing Call Parameters

We recorded the advertisement calls of frogs in the field. After detecting a frog with an infrared lamp, we turned off the lamp and slowly approached it so as not to disturb its calling behavior. We then recorded the calls of the target frog for 5 min using a super-directional microphone (ZOOM F1-SP, Tokyo, Japan) from a location about 1 m away from the target frog. We measured the chorus size at a radius of 5 m to obtain consistent results using the same method as previous studies [15,20]. After recording the calls, we counted the number of frogs calling within a 5 m radius and measured the chorus size, which can indicate the density of male frogs. We also conducted the survey while moving linearly to prevent the choruses from overlapping. Once the chorus size was measured, we passed by more than 10 m and re-sampled. We also did not sample on the way back from the survey site that we had passed once. We then collected the frogs whose calls were recorded, wearing sterile gloves. If the frog escaped from the location before 5 min of recording, if we failed to collect the frog, or if the chorus size of the frog could not be accurately measured, we deleted the recorded sound and found a new frog to record the call and measure the chorus size.

**Figure 2:**
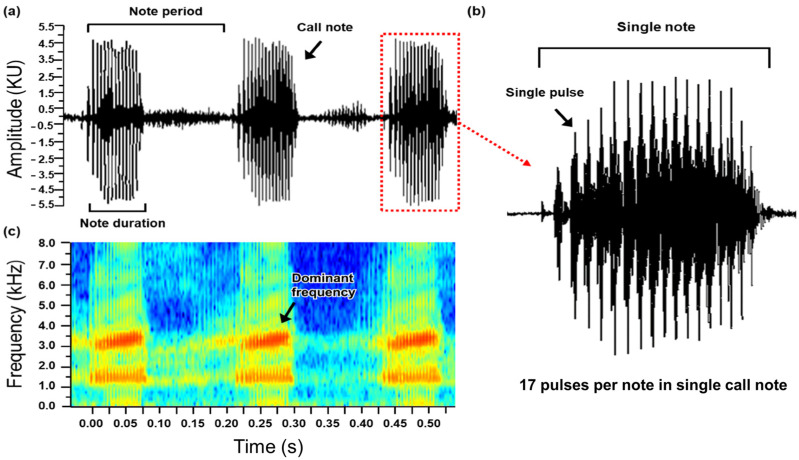
Oscillogram and sound spectrogram of advertisement calling from Japanese tree frogs. (**a**) oscillogram consisting of three call notes. Here we display the call note, note duration, and note period used to calculate call rate. (**b**) oscillogram consisting of single call note. Here we display a single pulse used to determine pulse per note. (**c**) Sound spectrogram composed of three call notes. Here we can see the dominant frequency of the advertisement calling.

The recorded calls were analyzed using Raven Pro software version 1.6 (Cornell Laboratory of Ornithology, Ithaca, NY, USA). We analyzed four parameters in the frog calls (Figure 2): (1) dominant frequency: the frequency with the highest energy in the call note; (2) pulses per note: the number of pulses in one note; (3) note duration: the length of a note (beginning time of note-end time of note); and (4) call rate: the speed of the call (1/note period (note-to-note spacing)). For all parameters, we used the average value of 20 call notes per individual.

### 2.3. Saliva Extraction and Hormone Assay

The frogs’ saliva was collected and extracted for the analysis of corticosterone, a hormone produced in response to glucose metabolism, and testosterone, a male sex steroid hormone. Because corticosterone can increase rapidly within 3 min following acute stress [24], we extracted saliva within 3 min of collecting the individual. First, we cut Salivary Cotton Pads (PURENAIL, Gillingham, Kent, UK) into 10 mm × 10 mm squares using sterile scissors, and then weighed each cotton swab to the nearest 0.001 using a digital scale. We then added this to a 1.5 mL microcentrifuge tube and recorded the weight on the tube. The mouth of the frog was gently opened using a sterile pipette tip, and a pre-weighed dry cotton swab was applied to the frog for 1 min to extract saliva. The extracted saliva was then transferred into a 1.5 mL microcentrifuge tube and kept in an icebox maintained at 4 °C in the field. Individuals with foreign substances on the cotton swab, such as blood or dirt, and individuals whose saliva extraction process was delayed for over 3 min after collection were excluded from the study. These animals continued to be held at one site until all animals had been sampled to prevent recapture and were then released prior to returning to the lab.

Swabs used for saliva extraction were weighed to the nearest 0.001 g using an electronic balance. By subtracting the previously weighed value, the exact mass of saliva obtained was calculated. After weighing, the swabs were stored in a freezer at −40 °C for 2 weeks. After 2 weeks, the swabs were transferred into a perforated microcentrifuge tube, and 150 μL of ELISA buffer was added, then centrifuged at 5000× *g* for 10 min. The supernatant was extracted, and a protocol for removing interfering proteins was applied. In our previous study, it was shown that interfering proteins in saliva interfere with the analysis of corticosterone and testosterone in Japanese tree frogs [15]. Therefore, referring to previous our study, we used trichloroacetic acid (TCA) to remove interfering proteins [15,25]. To 10 mg of saliva, we added 1.5 μL of TCA; then we centrifuged at 3000× *g* for 8 min to precipitate the proteins and separated the supernatant. The levels of the hormones were then measured using a 96-well corticosterone ELISA kit (501320, Cayman Chemical, Ann Arbor, MI, USA) and a 96-well testosterone ELISA kit (582701, Cayman Chemical, Ann Arbor, MI, USA) following the manufacturers’ instructions. In both cases, absorbance was read by a microplate spectrophotometer at 412 nm, and the concentration of these hormones was determined based on the weight of saliva.

### 2.4. Analysis of Bd Infection Load

After saliva extraction was completed in the field, frogs were used to determine their *Bd* infection load. After rinsing the individuals once with 50 mL of sterile distilled water to remove debris, we swabbed them eight times on the dorsal, eight times on the ventral, and twice from the legs to the toes using Isohelix DNA Buccal Swabs (SK-2S, Isohelix, Harrietsham, UK). Sterile nitrile gloves were changed each time a new individual was collected to minimize the possibility of cross-contamination. Then, we put only the head of the swab into a 2 mL microcentrifuge tube included in the swab kit and stored in an icebox at 4 °C in the field. Swabs were subsequently transported to the Animal Laboratory at Gongju National University and immediately used for microbial DNA extraction. PrepMan Ultra Sample Preparation Reagent (Applied Biosystems, Waltham, MA, USA) was used to extract the genomic DNA of the microorganisms from the swabs according to the manufacturer’s instructions. The extracted DNA was used to perform quantitative PCR (qPCR) to determine the *Bd* load per sample. The extracted genomic DNA samples were stored at −20 °C until analysis.

For the real-time quantitative PCR (qPCR), referring to the previously described protocol, the primer ITS1-3 Chytr, 5.8S Chytr and the TaqMan probe Chytr MGB2 were used to detect ITS 1-5.8S-2 rRNA genes from the obtained genomic DNA [26]. TaqMan qPCR was performed in duplicate in 20 µL volumes using SsoAdvanced Universal Probes Supermix (Bio-Rad Laboratories, Hercules, CA, USA) and was run in three steps on a Bio-Rad CFX Duet (Bio-Rad Laboratories, Hercules, CA, USA) for all samples, including positive and negative controls (distilled water). The positive control consisted of *Bd* DNA obtained from the Fire-bellied toad (*Bombina orientalis*). Preincubation was performed at 95 °C for 10 min, followed by 45 cycles of annealing at 95 °C for 15 s and extension at 60 °C for 1 min. Triplicate repeats of positive control of the *Bd* gene and negative controls were used to determine the infection load of the samples. The results of the real-time quantitative PCR were analyzed using Bio-Rad CFX Manager v1.6 (Bio-Rad Laboratories, Hercules, CA, USA) provided by Bio-Rad. A Cq value of 39 or less was determined to be positive according to the criteria for chytrid positivity published by the World Organization for Animal Health (WOAH) [27].

In this study, the positive control was a 3165 bp DNA fragment with a concentration of 12.3 ng/µL. The molecular weight of the 3165 bp DNA fragment was calculated as 2,088,900 g/mol. Using this molecular weight and DNA concentration, the number of copies per µL was calculated as 3.55 × 10^9^ copies/µL. The reaction volume was 20 µL, giving a total of 3.55 × 10^9^ × 20 copies in the reaction. In calculating *Bd* load, the relative quantification method was assumed with 100% PCR efficiency. The *Bd* concentration was calculated using the following formula:

Bd Load (ng/µL) = Total copies in reaction of positive control/2^−(Cqsample−Cqpos)^

where:

C: The concentration of the positive control (12.3 ng/µL)

Cqsample: The Cq value of the sample

Cqpos: The Cq value of the positive control (4.45)

This formula is based on the assumption that for each 1-cycle increase of the Cq value, the DNA concentration decreases with a factor of 2-a decrease corresponding to an ideal or 100% amplification efficiency. Infection status was determined as a threshold Cq value of 39. Samples with Cq values ≤ 39 were classified as “Infected”, while samples with higher or undetectable Cq values were considered “Not Infected”.

### 2.5. Blood Extraction and Immune Assay

Swabbed individuals were temporarily stored in sterile conical tubes and transported to the animal laboratory of Kongju National University within 30 min. Blood was extracted by cardiac venipuncture within 2 h of transport. Blood was drawn via a cardiac venipuncture using a sterile 31G Insulin Syringe (BD Ultra-Fine, Franklin Lakes, NJ, USA) The blood volume collected from the frogs was approximately 7% of the individual’s body weight, following previously published safe blood collection standards for terrestrial amphibians [28]. Then, collected blood carefully transferred into a heparinized tube from the syringe. Afterwards, blood was transferred from the capillary tube to a 1.5 mL microcentrifuge tube using a pipette and left at room temperature for 1 h to allow sufficient reaction between blood and heparin. The reacted blood was centrifuged at 3000× *g* for 5 min, and the supernatant was extracted to obtain plasma. If hemolysis was found in the extracted plasma, the entire set of samples from the individual was removed. In other words, not only was the blood excluded from the experiment, but also the recorded calls and extracted saliva. Individuals not used for analysis were released the next day at the original collection site.

We analyzed bacterial killing ability (BKA), one of the most popular innate immunity indicators in ecological immunology analysis. This indicates the immune status in response to complement response [29,30]. After obtaining intact plasma for analysis, we performed an analysis of the BKA of the plasma. We diluted the plasma 20× by adding 10 μL of plasma to 190 μL of amphibian ringer solution (ARS). Then, 10 μL of a non-pathogenic *Escherichia coli* (Microbio-Logics #24311-ATCC 8739, St. Cloud, MN, USA) working solution (approximately 10^6^ microorganisms) was added to the plasma diluent. A positive control was prepared by mixing 10 μL of the *E. coli* working solution with 200 μL of ARS, and 210 μL of ARS was used as a negative control. All controls and mixtures were incubated at 37 °C for 60 min, and then 500 μL of tryptic soy broth (TSB) was added. The suspension was then transferred to a 96-well plate in duplicate (300 μL each). Plates were incubated at 37 °C for 2 h and measured six times at 1-h intervals on a microplate spectrometer (wavelength 600 nm). BKA was assessed during the bacterial growth phase and was calculated using the formula: [1 − (optical density of sample/optical density of positive control)], which represents the percentage of dead microorganisms in the plasma compared to the positive control.

### 2.6. Measurement of Sperm Quality

We used 1 mL cooled Amphibian Ringer Solution to suspend the testes, which were gently torn using pointed sterile forceps to release the contained sperm. Sperm quality, including sperm viability, deformity, and density, was measured in male frogs. Sperm viability was measured by adding 0.4% (*w*/*v*) Eosin Y Acid Red to the sperm suspension in a 1:1 (*v*/*v*) ratio for staining the sperm cytoplasm and examined under a 400× microscope after 30 s. Sperm showing red cytoplasm were considered dead, whereas translucent sperm cells were counted as live [31]. Sperm density estimates were made using a hemacytometer to count the true sperm density of the sperm suspension. A 10 μL sample of the sperm suspension was pipetted into the hemocytometer, and sperm in five secondary square grids were counted. The total sperm count was determined by multiplying the density estimate by the weight of the sperm suspension, assuming that 1 mL of sperm suspension weighs 1 g. To confirm morphological deformities in the sperm of male frogs, 10 μL from the sperm suspension was placed on a microscope slide and photographed at 400× magnification to analyze 100 sperm per individual.

In the current study, total deformed sperm were counted, and the percentage (%) of normal sperm was calculated. Sperm morphology was assessed based on abnormalities in sperm parameters: head, midpiece, and tail. A sperm was considered normal if it had a single head, few cytoplasmic droplets, and intact flagella [32]. Conversely, it was considered deformed if there was at least one of the following defects: head defects, including vacuolated head, amorphous head, twisted head, or double head; midpiece defects, including cytoplasmic droplets and irregularly shaped midpiece; and tail defects, including double tail and terminal droplet.

### 2.7. Statistical Analysis

Using the Mann-Whitney U-test we compared chorus size, physical condition (body length and body weight), sperm quality (vitality, deformity, and density), physiological states (innate immunity, corticosterone, and testosterone), and call parameters (dominant frequency, pulse per note, note duration, and call rate) were compared between infected and uninfected males. Non-metric Multidimensional Scaling (NMDS) was applied to investigate the multivariate relationships between the physiological and behavioral parameters of Japanese tree frogs. NMDS is a nonparametric ordination method that has been extensively used for reducing high-dimensional ecological data to a space of low dimensionality while maintaining the structure of distances or dissimilarities [33]. The dataset consisted of quantitative measurements of physiological parameters, such as BKA, CORT, testosterone, reproductive traits, including sperm density and vitability, and calling behaviors, including dominant frequency and call rate. Prior to analysis, all numeric variables were standardized (Z-score transformation) to make them comparable and reduce the effect of differing scales. NMDS analyses were performed using the vegan package in R version 4.2.2 [34]. Euclidean distance was selected based on the continuous nature of the data. By interpretability and the value of stress, two dimensions for ordination were chosen, k = 2. The starting configuration is random, and a maximum of 100 iterations was allowed to converge to an optimal solution. In the present case, stress has been used as a goodness-of-fit measure, and the lower the value of stress, the better the data are represented in the reduced dimensions.

## 3. Result

### 3.1. Bd Infection Intensity

Among 70 male frogs, 13 males were infected with chytrid fungus, and 57 males were not infected. Furthermore, the average the amount of *Bd* loaded on the infected frogs was 8.235 ± 5.99 ng/µL. None of the infected male frogs showed any external pathological symptoms.

### 3.2. Chorus Size and Physical Conditions

**Figure 3:**
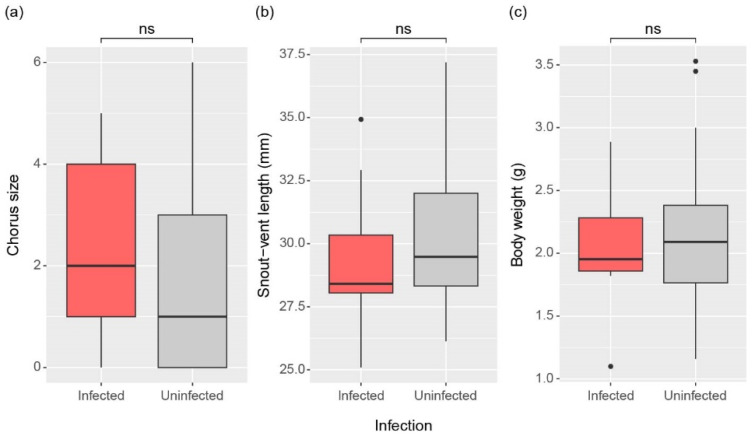
Comparison of (**a**) Chorus size and (**b**) Snout-vent length, (**c**) Body weight between infected and uninfected male frogs. The significance of differences was determined by the Mann-Whitney U test and is indicated for no significance (ns, *p* > 0.05). The top line of the box represents the third quartile, the bottom line represents the first quartile, and the thick horizontal line in the middle represents the median. The vertical lines represent the 1.5 interquartile range, and dots represent outliers.

There was no significant difference in chorus size between infected and non-infected individuals (W = 458, *p* = 0.179; Figure 3a). Additionally, there was no significant difference in snout-vent length, representing body length (W = 295, *p* = 0.261), or in body weight (W = 372, *p* = 0.988) between the two groups (Figure 3b,c).

### 3.3. Sperm Quality

**Figure 4:**
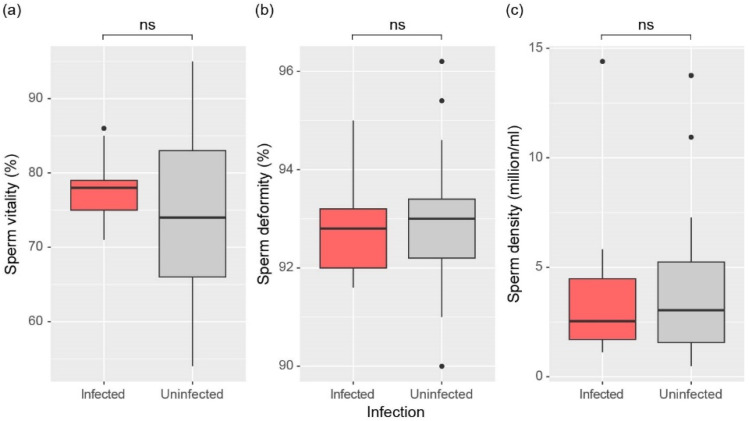
Comparison of (**a**) Sperm vitality, (**b**) Sperm deformity, and (**c**) Sperm density between infected and uninfected male frogs. The significance of differences was determined by the Mann-Whitney U test and is indicated for no significance (ns, *p* > 0.05). The top line of the box represents the third quartile, the bottom line represents the first quartile, and the thick line in the middle represents the median. The vertical lines represent the 1.5 interquartile range, and dots represent outliers.

The Mann-Whitney U test showed no significant differences in any parameters of sperm quality between infected and uninfected males (Figure 4a–c). Specifically, we found no significant differences in sperm viability (W = 431, *p* = 0.360), sperm deformity (W = 355, *p* = 0.820), or sperm density (W = 342, *p* = 0.672) between the infected and non-infected groups.

### 3.4. Physiological States

**Figure 5:**
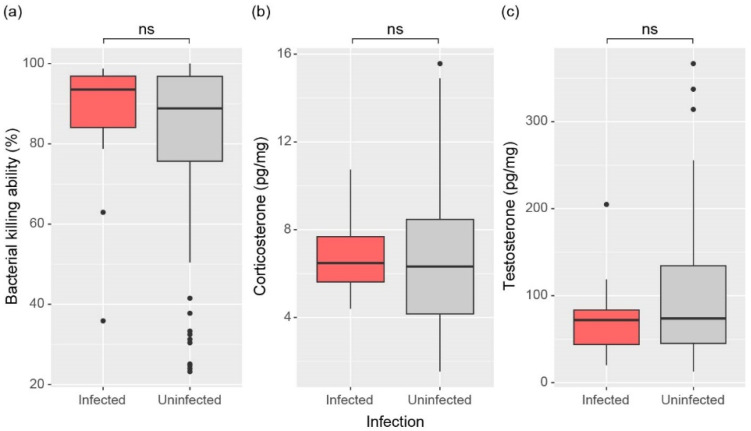
Comparison of (**a**) Bacterial killing ability, (**b**) Corticosterone, and (**c**) Testosterone between infected and uninfected male frogs. The significance of differences was determined by the Mann-Whitney U test and is indicated for no significance (ns, *p* > 0.05). The top line of the box represents the third quartile, the bottom line represents the first quartile, and the thick line in the middle represents the median. The vertical lines represent the 1.5 interquartile range, and dots represent outliers.

Similar to the previous results, physiological states showed minimal differences between *Bd*-infected and uninfected males. BKA, an indicator of innate immunity, appeared lower in uninfected males, though the difference was not statistically significant between the two groups (W = 426, *p* = 0.406; Figure 5a). Likewise, corticosterone, related to glucose metabolism (W = 404, *p* = 0.618), and testosterone, a male sex hormone (W = 320, *p* = 0.450), showed no significant differences between the groups (Figure 5b,c).

### 3.5. Call Parameters

**Figure 6:**
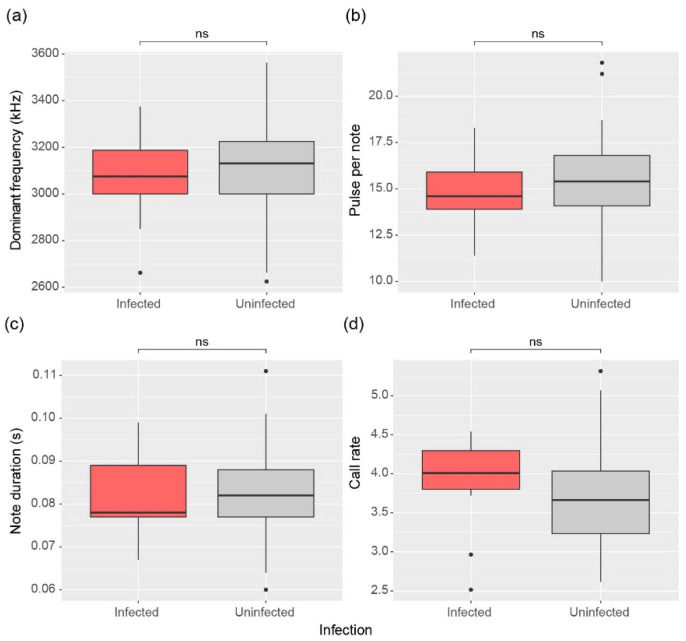
Comparison of (**a**) Dominant frequency, (**b**) Pulse per note, (**c**) Note duration, and (**d**) Call rate between infected and uninfected male frogs. The significance of differences was determined by the Mann-Whitney U test and is indicated for no significance (ns, *p* > 0.05). The top line of the box represents the third quartile, the bottom line represents the first quartile, and the thick line in the middle represents the median. The vertical lines represent the 1.5 interquartile range, and dots represent outliers.

Patterns of advertisement calling in frogs showed consistent results, with no significant differences between *Bd* infection groups. Dominant frequency (W = 366, *p* = 0.952), pulses per note (W = 313, *p* = 0.389), and note duration (W = 351, *p* = 0.780) did not differ significantly between infected and uninfected males (Figure 6a–c). On the other hand, call rates appeared to differ subtly, but ultimately there was no statistical difference (W = 494, *p* = 0.063) (Figure 6d).

### 3.6. Relationship of Behavior and Physiological Components

**Figure 7:**
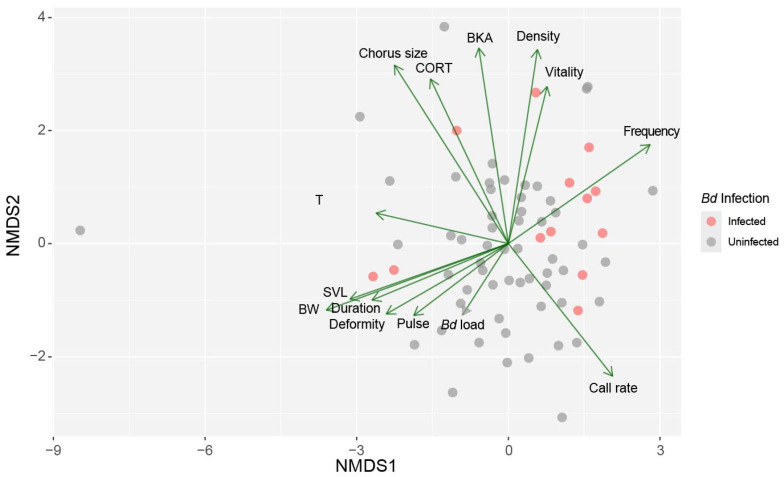
Non-metric multidimensional scaling (NMDS) ordination showing a high level of overlap between infected and uninfected frogs, but also subtle trends with some specific components: *Bd* infection load, chorus size, snout-vent length (SVL), body weight (BW), sperm vitality (vitality), sperm deformity (deformity), sperm density (density), bacterial killing ability (BKA), corticosterone (CORT), testosterone (T), dominant frequency (frequency), pulse per note (pulse), note duration (duration), call rate.

NMDS was conducted to explore the relationships among *Bd* infection status, physiological states, reproductive traits, and calling behaviors. The final NMDS, with a stress value of 0.08, yielded a reliable two-dimensional representation of the data. The NMDS ordination shows the high degree of overlap among *Bd*-infected and uninfected frogs, demonstrating a very minimal overall separation between the two. Despite the general trend observed, it becomes clear that subtle trends exist in the directionality of particular traits (Figure 7).

Variables representing BKA, sperm density, sperm vitability, and dominant frequency were closer to infected individuals, while uninfected individuals had a stronger relationship with higher body weight, SVL, note duration, sperm deformity, and pulse per note (Figure 7). Meanwhile, sperm density and sperm vitability were found to have negative correlations with *Bd* load, while body weight and SVL had inverse relationships with dominant frequency. Chorus size, corticosterone, and BKA appeared to have negative correlations with call rate (Figure 7).

**Figure 8:**
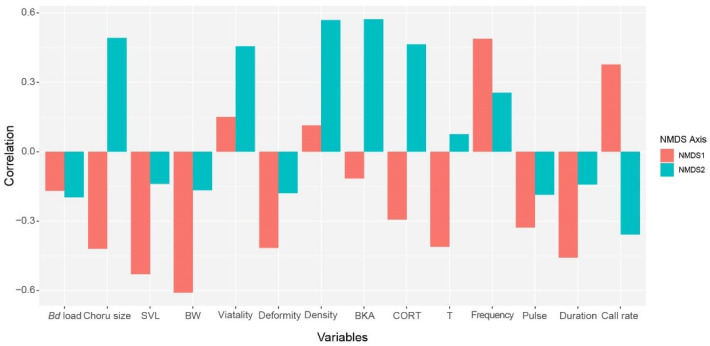
The variable correlation analysis from non-metric multidimensional scaling (NMDS) showing the pattern of the association of *Bd* infection load with chorus size, body condition, sperm quality, physiological status, and call sound components.

The variable correlation analysis also lends credence to these facts. On the NMDS1 axis, call rate (R = 0.46) and dominant frequency (R = 0.51) had positive loadings, whereas body weight (R = −0.61), SVL (R = −0.53) and sperm deformity (R = −0.42) had negative loadings (Figure 8). Meanwhile, in the NMDS2 axis, chorus size (R = 0.49), corticosterone (R = 0.46), BKA (R = 0.57), sperm vitality (R = 0.47), and sperm density (R = 0.57) showed a strong positive relationship, whereas call rate (R = −0.36) had negative relation (Figure 8).

## 4. Discussion

Our study aimed to quantify behavioral and physiological responses to *Bd* infection in Japanese tree frogs from Korea, a species known for its resistance to *Bd* and to explore the implications for population sustainability. Results showed no significant differences between infected and uninfected males in terms of chorus size, body size (length and weight), sperm quality (vitality, deformity, and density), innate immunity (BKA), physiological markers (corticosterone and testosterone), or call parameters (dominant frequency, pulses per note, note duration, and call rate). This outcome aligns with previous research, which has shown that species with low susceptibility to *Bd* exhibit minimal immune and physiological responses to infection compared to highly susceptible species [9,35,36]. Therefore, lack of differences in simple comparisons is expected to be due to differences in *Bd* resistance among populations rather than a low infection load in this population.

### 4.1. Increased Reproductive Investment in Response to Bd Infection

In particular, we referenced previous findings indicating that *Bd* infection can increase call rates in frogs [14]. Since this previous study involved the same species as ours, we anticipated similar results. The increase in calling effort may enhance attractiveness to females, thereby potentially boosting reproductive success [37,38]. However, improper energy trade-offs can sometimes reduce individual fitness [15,39,40]. We thought it was important to test whether this could reduce physiological abilities related to reproductive success, such as sperm quality; because the increase in reproductive effort due to Bd is not more likely to be evidence of healthier males than a sign to be a trade-off that causes pathogen-infected frogs to divert energy investment from later life history or other physiological traits [17]. If sperm quality is reduced, this would mean that, in addition to directly affecting individual mortality, the pathogen could indirectly contribute to long-term population decline through inappropriate energy trade-offs. However, our results did not find an inappropriate energy balance due to *Bd* infection in resistant species.

No significant differences were observed in *Bd* infections, but NMDS showed that infected individuals had slightly higher sperm density and vitality. The same pattern was observed in *Bd* load. Thus, the energy used to resist or eliminate *Bd* in *Bd*-resistant Japanese tree frogs does not appear to be associated with decreased sperm quality. A previous study found that two species with *Bd* sensitivity had more developed testes and ovaries, as well as higher amounts of germ cells following infection [41]. We show that reproductive investment and reproductive effort are more evident in *Bd*-susceptible species, but that subtle trends may also emerge in *Bd*-tolerant species.

### 4.2. Relationship in Behavioral and Physiological Components by Bd Infection

We found some important trends between the components. In uninfected individuals, there was a positive relationship with body length and body weight. This appears to be a result of the fact that individuals with smaller body sizes are generally more likely to be infected [42]. On the other hands, the dominant frequency was negative related the body weight and size, and associated with *Bd*-infected individuals. Larger body size is associated with lower dominant frequency of calling, which is a universal finding in frogs [20]. As a result, infected individuals associated with small body size may have a higher predominant frequency. This larger body size was associated with reduced sperm deformity, a pattern similarly documented in earlier studies [43]. Males get larger with age but face a natural decline in sperm quality. This decline may, however, be offset by the improvement in their overall lifetime reproductive success promoted by experience and established status in the breeding hierarchy [44]. Such a suggestion might imply that male reproductive quality cannot be gauged by sperm vitality alone but also by mating tactics, sperm quantity, and female mate choice.

On the side, chorus size is associated with lower call rate and higher corticosterone. Chorus size is an indicator of male density, and generally, higher male density can lower call rates, which helps males conserve energy, allowing them to call longer or promote themselves more effectively [45,46]. Additionally, higher chorus size increases ecological risks, such as predation [47]. Previous studies have found that *Bd* infections peak during periods of low stream flow when frogs are concentrated in drying pools [48]. Similarly, our study sampled frogs during the breeding season when crowding occurs, which may have contributed to this effect that high *Bd* infection induces high density of these males. Close-range vocal interactions are also associated with increased corticosterone [49]. Corticosterone level is a hormone that regulates glucose metabolism, and when increased, it can help mobilize energy and help individuals cope more quickly with stressful situations [50,51]. Likewise, high corticosterone level may be important at large chorus sizes because it triggers faster responses in male competition, allowing faster acquisition of females.

The most important relationship is the positive relationship between BKA and *Bd*-infected individuals. Contrary to our expectations, a high infection load of *Bd* was associated with increased innate immunity. This may be due to immune activation following *Bd* infection. Previous studies have reported lowered immunity following *Bd* exposure in Pacific tree frogs (*Pseudacris regilla*), a species with catastrophic *Bd*-induced mortality [36]. Interestingly, in Cascades frogs (*Rana cascadae*), where there was no *Bd*-induced mortality, innate immunity was upregulated within 48 h of *Bd* exposure [36]. Thus, we expect this result to be a consequence of collecting them when their immunity was already heightened by the infection. They may show high immune activation, especially since the target species is resistant to *Bd*.

### 4.3. Population Sustainability in Species with Resistance to Bd

This important finding—that *Bd*-resistant species show immune enhancement in response to *Bd* infection load and no impairment in sperm quality—supports existing studies suggesting that *Bd* infection may actually have a positive effect on population maintenance [18,19]. Previous studies have shown that populations of southern Darwin’s frogs, which are catastrophically affected by *Bd*, increased in population size due to increased reproductive effort despite high mortality [19]. This might indicate that even if there is a population decline following an initial infection in a species that is not *Bd*-resistant, this can be a compensatory mechanism for the population if it subsequently develops *Bd* resistance and is able to successfully coexist with *Bd*.

On the other hand, it is known that *Bd* increases reproductive effort because the host perceives it as a threat of death [14,16]. Similarly, in species that are resistant to *Bd*, infection may not have a lethal effect, but it may still be enough to make the host uncomfortable, potentially increasing reproductive investment. Notably, this *Bd* susceptibility and response may vary not only among species but also within populations of a species [19]. Populations or species with relatively high susceptibility may exhibit stronger reproductive investment, even if they are *Bd*-resistant. On the other hand, in species or populations that have coexisted with *Bd* for a long time and have developed an immune system, as in our study, this response may be very weak or nonexistent. Understanding reproductive investment as a function of *Bd* susceptibility will surely help us predict and manage the impact of *Bd*, a large-scale epidemic in amphibians, on the sustainability of populations.

## 5. Conclusions

Our study offers hope for addressing the global decline of amphibians due to *Bd* strains. By systematically managing populations that decline due to the disease, if they recover from or adapt to the disease and stabilize, it is possible that declining populations can recover or even increase. On the other hand, even if a species’ population itself does not decline due to the disease, it can still influence other species by acting as a *Bd*-resistant reservoir species [52,53]. While most species in Korea have immune systems resistant to *Bd* and may not be significantly affected, this could be a problem on other continents. If populations of *Bd*-resistant species increase in response to disease impact, the abundance of *Bd* in local amphibian communities may increase, potentially affecting non-resistant species. Therefore, we suggest that future studies should be conducted at the community level to determine the abundance of *Bd* and the susceptibility of different species.

## Data Availability

Data are contained within the article.
